# Use of the Behaviour Change Wheel to design an intervention to improve the provision of mental wellbeing support within the audiology setting

**DOI:** 10.1186/s43058-023-00427-1

**Published:** 2023-05-02

**Authors:** Rebecca J. Bennett, Romola S. Bucks, Lisa Saulsman, Nancy A. Pachana, Robert H. Eikelboom, Carly J. Meyer

**Affiliations:** 1 Ear Science Institute Australia, 1 Salvado Road, Subiaco, WA Australia; 2 Centre for Ear Sciences, The University of Western Australia, Perth, Australia; 3 School of Health and Rehabilitation Sciences, The University of Queensland, St Lucia, Australia; 4 Medical School, Curtin University, Bentley, Australia; 5 The Raine Study, School of Population and Global Health, The University of Western Australia, Perth, Australia; 6 School of Psychological Science, The University of Western Australia, Perth, Australia; 7 School of Psychology, The University of Queensland, St Lucia, QLD Australia; 8 Department of Speech Language Pathology and Audiology, University of Pretoria, Pretoria, South Africa; 9 Department of Clinical, Educational and Health Psychology, University College London, London, UK

## Abstract

**Objective:**

This study describes the development of an intervention to increase the frequency of audiologists’ asking about and providing information regarding mental wellbeing within adult audiology services.

**Design:**

The Behaviour Change Wheel (BCW), an eight-step systematic process, was followed to develop the intervention. Reports describing the first four steps are published elsewhere. This report describes the final four steps and details the intervention developed.

**Results:**

A multifaceted intervention was developed to change audiologists’ behaviours relating to providing mental wellbeing support to adults with hearing loss. Specifically, three behaviours were targeted: (1) asking clients about their mental wellbeing, (2) providing general information on the mental wellbeing impacts of hearing loss, and (3) providing personalised information on managing the mental wellbeing impacts of hearing loss. A variety of intervention functions and behaviour change techniques were incorporated into the intervention, including instruction and demonstration, information about others approval, adding objects to the environment, use of prompts/cues, and endorsement from credible sources.

**Conclusion:**

This study is the first to use the Behaviour Change Wheel to develop an intervention targeting mental wellbeing support behaviours in audiologists and confirms the usability and usefulness of the approach in a complex area of clinical care. The systematic development of the Ask, Inform, Manage, Encourage, Refer (AIMER) intervention will facilitate a thorough evaluation of its effectiveness in the next phase of this work.

**Supplementary Information:**

The online version contains supplementary material available at 10.1186/s43058-023-00427-1.

Contributions to the literature

- Published examples of the use of behaviour change theory to guide development of complex health interventions contribute to the ongoing development and refinement of models and their application in future research. We report an example of the development of a complex health intervention guided by the Behaviour Change Wheel (BCW) process, highlighting the benefits of empowering healthcare professionals to take a more active role in the holistic healthcare needs of their clients.
- The mental wellbeing impacts of hearing loss are not generally addressed within routine audiological care. We demonstrate use of the BCW for the development of an intervention with the aim of increasing the frequency of audiologists’ (i) asking clients about their mental wellbeing, (ii) providing general information on the mental wellbeing impacts of hearing loss, and (iii) providing personalised information on managing the mental wellbeing impacts of hearing loss, within adult audiology services.
- We also demonstrate how the inclusion of a pilot study in the development process facilitated further refinement of the intervention prior to a larger trial.

## Introduction

Acquired hearing loss is a highly prevalent chronic health condition with numerous social and emotional impacts for both the person with hearing loss and their significant others [1–3]. Hearing loss impairs communication and thus can inhibit the ability to socialise in meaningful ways. As hearing loss gradually worsens, people may adopt maladaptive coping behaviours, such as avoiding social activities or withdrawing during social interactions [3]. Due to a combination of the hearing and communication challenges imposed by hearing loss and also any maladaptive coping strategies acquired over years of hearing decline, adults with hearing loss are at increased risk of experiencing poor mental wellbeing, including social isolation [4], loneliness [5], anxiety [6], and depression [7].

The mental wellbeing impacts of hearing loss are not consistently addressed within routine audiological care [8]. Hearing rehabilitation services often focus on provision of hearing devices, rather than an individual’s lived experience of their disability [9]. Although hearing devices improve hearing sensitivity, and to some degree hearing handicap [10], they do not fully address the mental wellbeing impacts of hearing loss [7, 10–13]. Therefore, maladaptive coping behaviours may persist even with improved hearing function [3]. While some adults with hearing loss develop helpful coping strategies (such as controlling the listening environment, humour, acceptance, assertiveness, communication repair strategies, and accepting support from significant others), many lack effective coping strategies and may continue to experience negative mental wellbeing impacts following the adoption of hearing devices [3].

Audiologists could play a critical role in facilitating timely access and support for the mental wellbeing needs of adults with hearing loss. Audiologists are frequently placed in the role of counsellor as adults with hearing loss often raise emotional concerns associated with hearing loss and audiological rehabilitation [14–16]. Furthermore, research shows that adults with hearing loss feel better supported and are more likely to engage with their hearing rehabilitation when their audiologist acknowledges their state of mental wellbeing [14, 17]. However, research shows that audiologists rarely enquire about mental wellbeing [8, 18, 19] nor provide appropriate support to address wellbeing concerns when they are raised [14]. Barriers to psychological symptom detection and support are extensive and include lack of skill, knowledge, time, organisational support, and resources [8, 20]. Consumers and professionals are increasingly calling for targeted interventions that help address the mental wellbeing impact of hearing loss [18, 19, 21, 22].

Our previous research revealed that adults who experience social challenges and emotional distress as a result of their hearing loss not only want but expect their audiologist to discuss and provide support for mental wellbeing in the context of hearing loss and hearing rehabilitation [21]. Importantly, audiologists have positive views towards supporting their clients in managing mental wellbeing and wish to broaden their skills in order to provide this support within the clinical setting [8, 18, 20, 21]. It is important to note that, unlike practising psychologists, audiologists are not formally trained or qualified to provide intensive therapeutic interventions for the purpose of treating individuals with mental health concerns. However, providing some degree of mental wellbeing support is within a audiologists scope of practice [23–25] and can include clinical approaches such as improving social engagement with technology, delivering information and training on how to manage the listening environment, communication and social skills training, facilitating peer and other professional support, providing emotional support, involving communication partners, empowering clients through self-efficacy training, and promoting client responsibility [8, 26]. Given the broad range of clinical approaches used by some audiologists to support the mental wellbeing needs of adults with hearing loss [26], the acceptability of these approaches to adults with hearing loss [27], yet the low use of these approaches within routine clinical care [8], interventions are needed to increase and improve how audiologists deliver mental wellbeing support within routine audiological service delivery.

### A theoretically driven behaviour change intervention

We set out to develop an intervention to increase the frequency of mental wellbeing support provided within routine audiology consultations. Rather than develop an intervention specific to a particular appointment type or procedure, we focussed our intervention on the broader audiological care setting aiming to help audiologists be more receptive to detecting and addressing their client’s wellbeing needs during any and every appointment. Given that the provision of mental wellbeing support is a clinical behaviour, we set out to develop a behaviour change intervention. Changing behaviour is difficult [28] as many factors can influence the uptake of an intervention and the success of implementation will depend on the barriers to and enablers of the behaviour to be changed [29]. Frameworks designed to assist the development of behavioural interventions propose the use of phased evidence-based approaches, employing both qualitative and quantitative investigations, and engaging stakeholders at every step of the process [30, 31]. To assist clinical researchers in the development of behaviour change interventions, a group of implementation researchers in the UK developed the Behaviour Change Wheel (BCW), an eight-step systematic process founded in psychological and organisational theory relevant to health behaviour change [32]. Resultant interventions take many forms, based on the specific needs and context of the behavioural problem. For example, interventions may include policy-level changes [33], educational resources [34], or digital technology development [35–37]. The BCW has been widely used to develop interventions that are both acceptable to users and effective in achieving their aims. These include interventions designed to optimise changes in health behaviours such as audiologists’ engaging in shared goal planning for adults with hearing loss [38, 39] and audiology clinic staff’s application of family centred practices [40]. As the application of the BCW varies according to the target behaviour(s), intended population and implementation setting, published examples of its use contribute to the ongoing development and refinement of the method. Accordingly, the purpose of this report is to describe the development of a behaviour change intervention, informed by the BCW, with the aim of increasing the frequency of audiologists asking about and providing information regarding mental wellbeing within adult audiology services.

## Methods

### Clinical context

In Australia, most clients engage with a hearing service provider when they are ready to address unmet hearing needs. If they choose to adopt a hearing device (e.g. hearing aids), they typically receive two or three appointments over a 3-month period, followed by annual check-up appointments [41]. A range of providers are involved in hearing services in Australia, such as hospitals, large multinational companies, small family practices, not-for-profit organisations, and university clinics. This study was embedded within a not-for-profit hearing service provider in a socially advantaged area. Based on eligibility criteria, people can access some funding support for hearing services (and hearing devices) from a range of sources, including government, private health insurers, or workers compensation; however, approximately half of hearing services provided are self-funded. As with other allied health services, participating audiologists, employed by our partner clinic, provided hearing care using standard audiological equipment, followed the same clinical protocols, reporting to the same manager, and working with the same patient-focused organisational values. None of the clinicians worked with sales targets or received commission.

### Intervention development

In March 2020, we commenced the eight-step systematic process of intervention development outlined by the BCW [32]. Each step was conducted by the research team (including researchers with expertise in audiology, psychology and implementation science) in partnership with four experts recruited from our clinical partner (three audiologists with clinical and managerial expertise and one manager with expertise in clinic administration processes). Our findings from steps 1 to 4 have been published elsewhere and describe the selection of the three target behaviours [21] and an in-depth exploration of the barriers and facilitators to the three target behaviours [19, 42]. Steps 5 to 8 are the focus of this report, wherein we describe development of a single multifaceted intervention that aims to address all three target behaviours simultaneously. Steps 1 to 4 are described briefly below to provide context.

Steps 1 to 3 involved (i) defining the problem in behavioural terms, (ii) selecting the target behaviour(s), and (ii) Specifying the target behaviour(s). These steps were achieved through a series of stakeholder workshops involving adults with hearing loss and their significant others, as well as hearing healthcare professionals (audiologists, reception staff, and managers) [21]. During these workshops, participants discussed the problem behaviour, defined as “Providing psychosocial and mental wellbeing support for adults with hearing loss”. The top behaviours voted by participants to be the most promising for a behavioural intervention were further specified to form the three behaviours targeted by our intervention: (1) asking clients about their mental wellbeing, (2) providing general information on the mental wellbeing impacts of hearing loss, and (3) providing personalised information on managing the mental wellbeing impacts of hearing loss.

Step 4 involved identifying what in the person(s) and/or environment(s) needed to change for the target behaviours to occur. This was achieved through a series of focus groups and semi structured interviews with audiologists exploring the barriers and facilitators influencing the three target behaviours using a framework called the COM-B model of behaviour change [19, 42]. The COM-B model recognises that barriers and facilitators of Behaviour change may relate to Capability (e.g. skills, knowledge), Opportunity (e.g. social influences, physical environment), or Motivation (e.g. beliefs, intentions, emotional responses, habitual responses). The model proposes that if a behaviour is not taking place, barriers in one or more of these areas need to be addressed. Where some studies have applied both the COM-B and the Theoretical Domains Framework frameworks, we chose to use the COM-B only for three reasons: (i) we did not want to be constrained by the Theoretical Domains Framework; the COM-B allows greater freedom to explore barriers and facilitators to behaviour change; (ii) given that we were focusing on three behaviours simultaneously, we felt it would be burdensome for participants to explore each of the 14 Theoretical Domains Framework domains for each behaviour, although we acknowledge that there is a chance that we might have missed an important driver of behaviour in making this pragmatic decision; and (iii) our focus was on designing a behaviour change intervention and there is clear guidance in the BCW to map COM-B barriers and facilitators to intervention functions. The following barriers were identified in relation to the three target behaviours of the intervention.

*Asking about mental wellbeing *[19]. All audiologists described firsthand experiences of discussing mental wellbeing within clinical encounters; however, audiologists tended to wait for the client to raise the topic of mental wellbeing impacts of hearing loss and rarely asked about clients’ wellbeing directly. The main barriers preventing audiologists from *asking* about mental wellbeing related to lack of knowledge and skills to ask about mental wellbeing (capability), lack of time and resources (opportunity), limiting beliefs about how clients might respond to being asked about wellbeing (motivation), limiting beliefs about the benefits of asking about emotions (motivation), and uncertainty about audiologists’ scope of practice (motivation) [19].

*Providing general information on the mental wellbeing impacts of hearing loss *[42]*.* Audiologists demonstrated a high level of understanding regarding the range of mental wellbeing impacts of hearing loss and appear motivated to provide general information to clients regarding mental wellbeing; however, most described not performing this behaviour routinely. Barriers to information sharing included lack of time and resources (opportunity), low confidence in their ability to accurately relay the information (motivation), concern that not all clients would be open to receiving this sort of information (motivation), and feeling unsupported by managers and colleagues (opportunity) [42].

*Providing personalised information on managing mental wellbeing *[42]*.* Audiologists were cognisant of the need to provide information on wellbeing management strategies and help-seeking options and demonstrated a general desire to do so. However, barriers preventing audiologists from routinely providing information on managing mental wellbeing related to limited knowledge regarding which clients require this information (capability), what sort of information to provide (capability), how to present and discuss the information (capability), and time pressures and lack of resources for providing this information (opportunity). Participants also described being inhibited by a lack of knowledge regarding who, when, and how to refer for psychological services (capability), as well as perceiving a lack of psychology services and practitioners skilled to meet the unique needs of adults with hearing loss (motivation) [42].

Although step 4 investigated the factors influencing the three target behaviours separately, from this point, we worked towards developing a single multi-factorial intervention program targeting all three behaviours simultaneously. The three behaviours naturally co-occur and are reliant on one another, and therefore, we felt the intervention would be most successful if all three behaviours were addressed in parallel.

Steps 5 to 8 were conducted between October 2020 and March 2021.

All 93 factors (barriers and facilitators) identified across all three target behaviours in the COM-B analysis (step 4) were reviewed. We selected those factors that were most frequently raised by participants and considered most necessary to address (flow on impacts) in order to change the target behaviours. Forty-eight factors were selected across the three behaviours; however, some factors were similar across behaviours and therefore, once merged, there were 19 factors that needed to be targeted in the intervention (Additional file 1).

*Step 5. Identify intervention functions*. Potentially relevant intervention functions were identified by linking the 19 context specific barriers and facilitators with intervention functions evidenced to result in changed behaviour provided by the BCW framework (e.g. education, persuasion, incentivisation, coercion, training, restriction, environmental restructuring, modelling, and enablement) [32]. These decisions were guided by the APEASE criteria (Acceptability, Practicability, Effectiveness, Affordability, Side-effects, and Equity) [32], to guide prioritisation of the intervention functions appropriate that were most affordable, practical within the context of our partner clinic, effective and cost effective, acceptable to clients and staff, and had limited side-effects.

*Step 6. Identify policy categories*. The seven policy categories outlined within the BCW (e.g. guidelines, fiscal measure, legislation) were mapped onto intervention functions and explored as potential pathways to deliver the intervention, again using the APEASE criteria to guide selection.

*Step 7. Identify behaviour change techniques.* A behaviour change technique is defined as the “active component of an intervention design to change behaviour” [32]. Based on substantial behaviour change literature, particular techniques or ways of changing behaviour are known to be responsible for bringing about change in a certain way [43]. The lead researcher (RJB) matched the intervention functions identified in step 5 to the extensive list of potential behaviour change techniques outlined in the BCT-Taxonomy guidelines (https://theoryandtechniquetool.humanbehaviourchange.org/tool) to produce a list of potential behaviour change techniques. The research group and clinical partners together took this list, and used the APEASE criteria to generate priorities based on their context-specific work environment and considering the appropriateness for the population, setting, and intervention format. This shorter list of potential behaviour change techniques was then appraised against the literature and those deemed least appropriate according to the APEASE criteria were excluded at this point, to achieve the final list of most appropriate behaviour change techniques.

*Step 8. Identify mode of delivery*. Drawing on data generated within steps 5, 6, and 7, the research team and clinical partners brainstormed the most appropriate mode(s) for delivering the behaviour change techniques given the population group and setting and selected preferred delivery modes using the APEASE criteria, ultimately developing a blueprint for the intervention. Discussion was informed by the “suggested solutions” put forward by audiologists participating in the analysis of barriers and facilitators to the target behaviours (step 4) [42].

The Template for Intervention Description and Replication (TIDieR) guideline [44] was used to describe the intervention (Additional file 2).

## Results

BCW steps 5 to 8 are presented here complete with the findings that led to the development of the AIMER (Ask, Inform, Manage, Encourage and Refer) intervention and implementation strategy.

*Step 5. Identification of intervention functions.* Based on the 19 barriers and facilitators selected from the COM-B analysis, six of the nine intervention functions were identified as possible options: education (increasing knowledge or understanding), persuasion (using communication to induce positive or negative feelings or stimulate actuion), training (imparting skills), environmental restructuring (changing the physical or social context), modelling (providing an example for people to aspire to or imitate), and enablement (increasing means/reducing barriers to increase capability). Incentivisation (creating an expectation of reward), coercion (creating an expectation of punishment or cost), and restriction (using rules to increase the target behaviour through reducing the opportunity to engage in competing behaviours) were deemed not acceptable to audiologists or practicable within the work environment. Potential intervention functions were mapped to the 19 barriers and facilitators selected from the COM-B analysis and preferred intervention function selected (Additional file 3).

*Step 6. Identification of policy categories.* The research team considered policy categories that might support the delivery of the intervention functions identified in step 5. When using the APEASE criteria, Regulation, Legislation, Fiscal, and Communication/Marketing Measures were deemed inapplicable (outside our control) or impractical (beyond the scope of the study). This left the policy categories of Environmental Restructuring, Guidelines, and Service Provision as appropriate means to support the intervention. Given the context of this research, use of Guidelines was restricted to the clinical protocols specific to our partner clinic.

*Step 7. Identification of behaviour change techniques.* Based on APEASE ratings and intervention context, behaviour change techniques were selected and are further detailed in Table 1.

**Table 1:** Components of the AIMER intervention, a multifaceted intervention addressing the three target behaviours: (i) asking clients about their mental wellbeing, (ii) providing general information on the mental wellbeing impacts of hearing loss, and (iii) providing personalised information on managing the mental wellbeing impacts of hearing loss

Intervention functions	COM-B components served by intervention functions	Target behaviour^a^	Behaviour change techniques to deliver intervention functions	Operationalisation of the behaviour change technique	Mode of delivery/implementation strategy
**Arm 1. Training, education, enablement and persuasion**					
Training	**Psychological capability.** Audiologists require knowledge of how to detect signs and symptoms for emotional and psychological distress	Ask	4.1 Instruction on how to perform behaviour	We provided information on the signs and symptoms of emotional and psychological distress in adults with hearing loss	**In-person workshop.** Information was provided as part of the online in-person workshop **Self-directed learning.** The workshop recording was made available after the training session
Training	**Psychological capability.** Audiologists require knowledge of how to ask about emotional well-being	Ask	4.1 Instruction on how to perform behaviour	We provided information on how to ask about emotional wellbeing and provided with examples of language and sentence structure	**In-person workshop.** Information was provided as part of the online in-person workshop **Self-directed learning.** The workshop recording was made available after the training session
Training	**Psychological capability.** Audiologists require knowledge of treatment/management options for emotional and psychological distress, and how to provide reliable information regarding funding and access for psychological services	Manage	4.1 Instruction on how to perform behaviour	We recorded a video of a local clinical psychologist describing various treatment/management options and pathways for emotional and psychological distress	**In-person workshop.** Information was provided as part of the online in-person workshop **Self-directed learning.** The workshop recording was made available after the training session
Training	**Psychological capability.** Audiologists require knowledge of how to refer for mental wellbeing support	Manage	4.1 Instruction on how to perform behaviour 6.1 Demonstration of the behaviour	We developed two videos recorded by (i) a local clinical psychologist and (ii) a local GP describing referral processes for psychological support in Australia We developed referral report templates to provide structure and language to help audiologists report on mental wellbeing needs. Audiologists were shown how to use the newly developed report templates and asked to practice generating reports with the new templates in their own time	**In-person workshop.** The videos were incorporated into the pre-workshop recorded material and also played during the in-person workshop The electronic referral templates were embedded into the patient management system used so that they could be accessed in the same way as the other report templates used by the clinicians. As part of the in-person workshop. audiologists were shown how to use the newly developed report templates. The importance of aligning wording in reports with the exact wording used by clients was emphasised Audiologists were asked to practice generating reports with the new templates in their own time
Training	**Psychological capability.** Audiologists require language skills for discussing mental health-related topics	Inform and manage	4.1 Instruction on how to perform behaviour 6.1 Demonstration of the behaviour	Prior to the training, audiologists were asked to put forward phrases that their clients have said that they found difficult to respond to (e.g. “*All my friends are dead now*”, “*I've got no one left*”). In partnership with clinical psychologists and audiologists, we developed a deck of training flashcards, each stating one of the "difficult" client statements followed by a few suggested responses that demonstrate empathy and understanding	**In-person workshop.** Audiologists were given a set of flashcards in their workshop packs. The flashcards and how to use them to promote development of language skills for discussion mental wellbeing was discussed within the in-person workshop **Self-directed learning.** Audiologists were encouraged to use the flashcards in their own time to continue practicing using language to discuss mental wellbeing
		Ask, inform, and manage	6.1 Demonstration of the behaviour 9.2 pros and cons	We demonstrated how one might (i) ask about mental wellbeing, (ii) provide information on the mental wellbeing impacts of hearing loss, and (iii) provide information on mental wellbeing treatment/management strategies. We invited the audiologists to discuss what was good and bad about the examples provided, and to devise their own scripts for how they would feel most comfortable asking these questions and providing this information in the future	**In-person workshop.** We demonstrated how to perform the three behaviours using role play. We also provided a list of possible wording for asking about mental wellbeing and provided purposefully developed clinical resources to assist with information provision **Self-directed learning.** The workshop recording was made available after the training session
Training	**Reflective motivation.** Audiologists need to develop confidence in their ability to ask about mental wellbeing and respond with empathy when clients describe their challenges	Ask and inform	8.1 Behavioural practice/rehearsal	We provided instruction on how to respond with empathy, and opportunity to practice responding to difficult statements put forward by clients	**In-person workshop.** We provided information on the definition of empathy, the benefits of using empathy and examples of how empathy can be expressed within audiological interactions. We delivered an activity wherein we listed common statements made by clients and had the audiologists work in small groups to discuss and document how they might respond empathetically. The audiologists spent time reading the flashcards to reinforce how they might respond with empathy in challenging situations **Self-directed learning.** The workshop recording was made available after the training session. Audiologists were encouraged to use the flashcards in their own time to continue developing their confidence in responding with empathy
Education	**Psychological capability.** Audiologists require knowledge of who to refer to for mental wellbeing support	Manage	4.1 Instruction on how to perform behaviour	We developed a list of local referral partners, including identification of those who specialise in psychological support for people with hearing loss	**In-person workshop.** Audiologists were presented with the list of local referral partners generated and how to locate the list from within the organisations document storage systems **Self-directed learning.** The workshop recording was made available after the training session. The clinical resources and where to locate them were included in one of the post-workshop videos
Education and persuasion	**Reflective motivation.** Audiologists need reassurance that clients are open to receiving information on the mental wellbeing impacts of hearing loss	Inform	6.3 Information about others’ approval	We developed an educational resource wherein adults with hearing loss described how they cope with the mental wellbeing impacts of hearing loss and how grateful they were to receive this information and support from their audiologist (https://www.youtube.com/watch?v=rmu4e4hNlKs)	**In-person workshop.** Audiologists viewed the video within the training session and then reflected as a group on how receptive clients are to these conversations within general clinical appointments **Self-directed learning.** The recording was emailed to staff after the training session
Education	**Reflective motivation.** Audiologists need reassurance that asking about and providing mental wellbeing support is within their scope of practice	Ask and manage	6.3 Information about others’ approval	We provided information on the Australian Scope of Practice guidelines, as well as the USA guidelines to emphasise that provision of wellbeing support is within current practice guidelines	**In-person workshop.** Information on Scope of Practice guidelines were provided **Self-directed learning.** The recording was emailed to staff after the training session
Enablement	**Social opportunity.** Audiologists require reassurance from their managers that provision of mental wellbeing support is a vital part of their service provision despite it not being a claimable service	Ask, inform, and manage	3.2 Social support (practical)	The Chief Operating Officer and clinical managers demonstrated their support for focussing clinical time on clients’ mental wellbeing needs	**In-person workshop.** The Chief Operating Officer and clinical managers attended the training workshop and stated their support for the research program and the benefits of addressing mental wellbeing needs within routine audiological care
Persuasion	**Reflective motivation.** Audiologists need to feel responsible for (i) asking about mental wellbeing, (ii) providing information on the mental wellbeing impacts of hearing loss, and (iii) providing information on mental wellbeing treatment/management strategies	Ask, inform, and manage	5.1 Information about health consequences	We developed a persuasive resource wherein adults with hearing loss described their personal experiences of living with hearing loss, how this impacted on their mental wellbeing and how they wish their audiologist had helped them to better understand and address these mental wellbeing impacts of hearing loss (https://www.youtube.com/watch?v=zQbM2NPmU_Y)	**In-person workshop.** Audiologists viewed the video within the training session and then reflected individually on how often they provide mental wellbeing support to their clients **Self-directed learning.** The workshop recording was emailed to staff after the training session
Persuasion	**Reflective motivation.** Audiologists require reassurance that clients are open to receiving information on mental wellbeing treatment/management options during audiological appointments	Inform and manage	9.1 Credible sources	To address audiologists’ insecurities regarding how clients would react to being given information on mental wellbeing, we developed a video of clients describing positive stories of how their audiologists helped them to gain an understanding of how wellbeing is impacted by hearing loss, how they are interested to learn more about psychological treatment/management options during audiological appointments, and that management strategies are effective (https://www.youtube.com/watch?v=rmu4e4hNlKs)	**Pre-workshop videos.** The persuasive video was incorporated into the pre-workshop video content **In-person workshop.** Audiologists viewed the video within the training session and then reflected as a group on how receptive clients are to these conversations within general clinical appointments. **Self-directed learning.** The workshop recording was emailed to staff after the training session
Persuasion	**Reflective motivation.** Audiologists need reassurance that GPs would react positively to receiving a referral from an audiologist regarding concerns for a client’s mental wellbeing	Manage	9.1 Credible sources	We developed a video interviewing three local GPs on their views regarding the role of the audiologist in detecting the mental wellbeing impacts of hearing loss, and how GPs and audiologists can work together to provide multidisciplinary care. Within these videos the GPs explicitly stated how they would be pleased to accept referrals from audiologists regarding the mental wellbeing needs of their clients	**In-person workshop.** Audiologists viewed the video within the training session **Self-directed learning.** The recording was emailed to staff after the training session
Persuasion	**Reflective motivation.** Audiologists need reassurance that psychologists have the skills required to address the psychological needs of adults with hearing loss seeking psychological support	Manage	9.1 Credible sources	We developed a video interviewing a local clinical psychologist describing the many ways that psychologists can support the mental wellbeing needs of adults with hearing loss	**In-person workshop.** Audiologists viewed the video within the training session **Self-directed learning.** The recording was emailed to staff after the training session
Persuasion	**Reflective motivation.** Audiologists need reassurance that psychologists are open to receiving referrals from them	Manage	9.1 Credible sources	We developed a video interviewing a local clinical psychologist, wherein the psychologist described the referral process and explained how audiologists’ referrals promote holistic and multidisciplinary care for adults with hearing loss We also generated a list of local psychologists with experience in working with adults with hearing loss and who had indicated that they were willing to accept referrals from audiologists so that our audiologists could confidently refer	**In-person workshop.** Audiologists viewed the video within the training session **Self-directed learning.** The recording was emailed to staff after the training session
**Arm 2. Environmental restructuring**					
Environmental restructuring	**Psychological capability.** Audiologists require knowledge of who to refer to and how to refer for mental wellbeing support	Manage	12.5 Adding objects to the environment	We developed a list of local psychological services, specifically those who indicated that they would accept referrals from audiologists, and that they had experience working with older adults and'/or adults with hearing loss. We also developed lists of local community groups and a list of local mental health support groups	**In-person workshop.** Audiologists were presented with the lists and shown how to locate the lists from within the organisations document storage systems **Self-directed learning.** The workshop recording was made available after the training session. A copy of the lists and where to locate them were included in one of the post-workshop videos
Environmental restructuring	**Social opportunity.** Audiologists require reassurance that clients will be receptive to them asking about mental wellbeing	Ask	12.5 Adding objects to the environment	We developed a clinical resource, an animated explainer video, wherein an adult with hearing loss describes his experience of hearing loss and how it impacts on his mental wellbeing. The purpose of the video was to help clients realise the link between hearing loss nd mental wellbeing, prime them in that audiologists are likely to ask about mental wellbeing, and encourage them to think about how their hearing loss might be affecting them (preparing them for the mental wellbeing conversation with the audiologist). By making the audiologists aware of the video and dissemination plan, we hoped audiologists would be reassured that clients were anticipating and prepared for being asked about mental wellbeing within audiology appointments (https://www.youtube.com/watch?v=8CXMeU6oECU)	The video is disseminated to clients via (i) emailed to all clients prior to their hearing assessment, (ii) posted on the clinic’s social media pages, and (iii) played on the clinic’s waiting room TVs **In-person workshop.** Audiologists viewed the video within the training session and then reflected as a group on how clients might respond to the video and what they might now be expecting from their first appointment
Environmental restructuring	**Physical opportunity.** Audiologists require clinical resources to assist with (i) asking about wellbeing, (ii) providing information on the wellbeing impacts of hearing loss, and (iii) providing information on wellbeing treatment/management strategies	Ask, inform, and manage	12.5 Adding objects to the environment	In partnership with the target audiologists, we developed a series of clinical tools, including (i) two discussion tools incorporated into the existing flip chart used to aid client discussion (one facilitating asking about and discussing wellbeing in relation to hearing loss; one facilitating discussion on the stepped care approach to mental wellbeing management); (ii) a series of three client information sheets (describing the “Emotional impacts of hearing loss”, “Social impacts of hearing loss”, and how “Hearing loss affects relationships”); and (iii) a client brochure to assist discussion and information provision relating to mental wellbeing management options (including what the management options entail, access and funding)	To **embed** the factsheets within the audiologists’ workflow, we altered the customer management software used by the clinic staff to set up an automated factsheet printing system. As the audiologists enter their case notes, they can select which factsheets they want printed (including these three new ones as well as all of the existing factsheets previously used by audiologists, but manually printed or photocopied) **In-person workshop.** Audiologists were presented with the lists and shown how to locate the lists from within the organisations document storage systems **Self-directed learning.** The workshop recording was made available after the training session. A copy of the lists and where to locate them were included in one of the post-workshop follow up videos **Ongoing support**. The organisations clinical protocols were updated to reflect incorporation of the new clinical resources into routine practice
Environmental restructuring	**Social opportunity.** Audiologists require reassurance from their managers that provision of mental wellbeing support is a vital part of their service provision despite it not being a claimable service	Ask, inform, and manage	12.2 Restructuring the social environment	The management team demonstrated their commitment to supporting the wellbeing needs of clients, by allowing us to update their (i) Clinical Procedures Documentation to describe the use of these new resources, emphasising the importance of considering client’s mental wellbeing needs within appoints, and (ii) internal training procedures	**In-person workshop**. To reinforce this message, the clinical managers addressed the staff highlighting that provision of mental wellbeing support is a vital part of their service provision despite it not being a claimable service, noting that the Clinical Procedures Documentation and internal training procedures had been updated to reflect this new way of working
Environmental restructuring	**Automatic motivation.** Audiologists need reminders/prompts to help them remember to ask clients about mental wellbeing	Ask	7.1 Prompts/cues 6.1 Demonstration of the behaviour	To improve remembering and habit forming for asking clients about their mental wellbeing, we added a question on wellbeing to the electronic client case history form and in the clinical case notes (1st appointment and annual recall appointment templates) We also modified the client goals template to promote inclusion of goals relating to mental wellbeing impacts of hearing loss. To **embed** the new goal setting template within the audiologists’ workflow, we altered the customer management software used by the clinic staff to set up an automated printing system. After the audiologist completes the goals within the data management system, they can now easily print the goals to assist discussion with the client	**In-person workshop.** We showed audiologists where the changes had been made, and demonstrated how to use the new questions within the regular work flow **Ongoing support.** Audiologists were sent bimonthly emails promoting specific aspects of the intervention, including how to use the new questions in the case history forms and the goals template
Environmental restructuring	**Social opportunity.** Audiologists need to feel supported by peers in their workplace	Ask, inform, and manage	3.2 Social support (practical)	Senior audiologists with advanced skills in providing mental wellbeing support were identified as mental wellbeing champions	Three members of the clinical team were identified as mental wellbeing champions **In-person workshop.** We advised the audiologists as to who the champions were nd how to contact them should they need support. The members of the research team with dual research and clinical expertise also made themselves available to answer any question throughout the duration of the project
**Arm 3. modelling**					
Modelling	**Social opportunity.** Audiologists need to see their managers and senior staff role modelling provision of mental wellbeing support	Ask, inform, and manage	9.1 Credible sources	After the in-person training day, senior and well-respected audiologists (including the mental wellbeing champions) from the partner clinic were engaged to record themselves describing which of the new clinical resources were their favourite to use and why, as well as providing a demonstration of how they use the resource(s) within their routine workflow	**Ongoing support.** Videos were distributed to audiologists in the months following the in-person workshop

^a^This column denotes which of the three target behaviours is being targeted by the intervention component: Ask = Behaviour 1. *Asking* clients about their mental wellbeing; Inform = Behaviour 2. Providing general *information* on the mental wellbeing impacts of hearing loss; and Manage = Behaviour 3. Providing personalised information on *managing* the mental wellbeing impacts of hearing loss

^b^ *ERIC* The Expert Recommendations for Implementing Change (ERIC) list is a compilation of implementation strategies (https://impsciuw.org/wp-content/uploads/2019/08/ERIC-Strategy-Handout.pdf)

The research team then developed a multifaceted intervention strategy simultaneously addressing all three target behaviours that incorporated individual behaviour change techniques and intervention components into one intervention program. This included specifying what was involved in each component, how it would take place, and where and who was involved. This process resulted in the AIMER intervention, a three-armed intervention including (1) an education, training, enablement, and persuasion arm; (2) an in-house environmental restructuring arm; and (3) a modelling arm. The research team further developed the intervention strategy by revising the research evidence for each intervention component and developing a detailed intervention proposal to present to the clinical partner team.

**Fig. 1:**
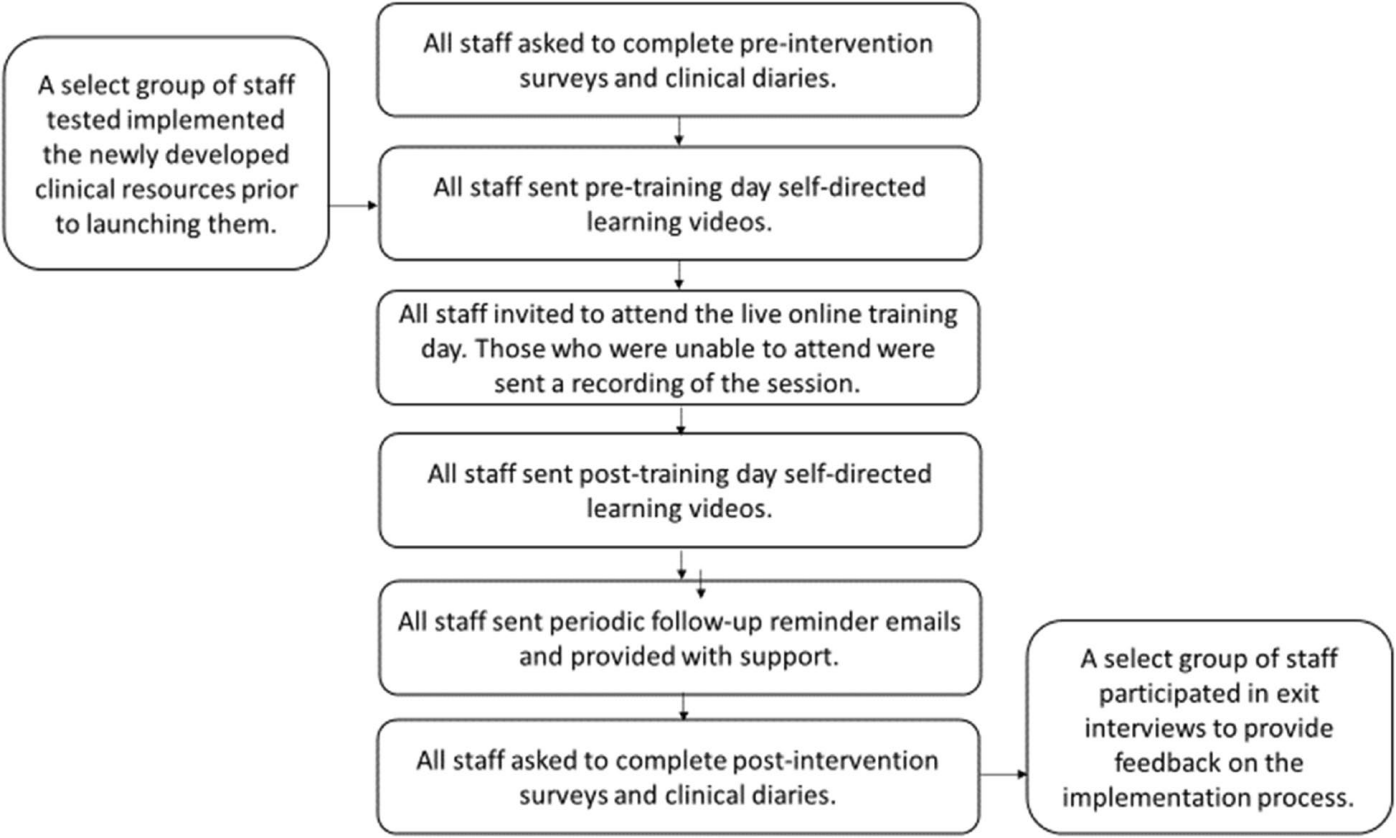
Depiction of how the audiologists received the AIMER interventional components and implementation evaluation measures (surveys and clinical diaries)

*Step 8. Intervention strategy and mode of delivery.* The research team and clinical partners together brainstormed how the potential intervention components might best be delivered within the specific context of our clinical partner organisation. The research team used these concepts to develop a detailed implementation strategy. However, the first draft of the implementation strategy required over 20 h of training and education and was thus deemed unfeasible. The research team and clinical partners used the APEASE criteria to refine the final configuration of the implementation strategy (Table 1). Implementation of the AIMER intervention program included (i) a 3-h hybrid in-person and online workshop; (ii) self-directed learning (including pre- and post-workshop videos and self-directed learning exercises, e.g. flashcards to develop vocabulary); and (iii) ongoing support (including access to champions and follow-up videos to reinforce workshop content) (Fig. 1). A train-the-trainer [45] approach was implemented so that internal trainers within the partnering clinic could provide in-house training and enablement for future employees.

### Description of the intervention

*Arm 1. Training, education, enablement, and persuasion* were designed to be delivered as a half-day in-person workshop supported by a series of self-directed activities, such as online educational videos provided before/after the in-person session. Due to continuing COVID-19 restrictions, the workshop was run as a live online event. Arm 1 intervention components were delivered across three 15-min videos provided to audiologists two weeks before the workshop; a 3-h live online workshop (to audiologists in clinic, office and home locations); and four 15-min videos delivered 2 weeks after the workshop. The workshop was delivered as a one-off group workshop. Training was primarily delivered by RJB a clinical and research audiologist with 15 years’ experience in training and education, as well as skills in counselling and provision of mental wellbeing support (Graduate Diploma in Counselling). Having worked with the partner organisation for 14 years RJB was well known to many of the staff participating in the intervention. All participating audiologists received a workshop kit prior to commencement of the workshop, which included a printed workbook to supplement learning during the workshop, a deck of flashcards, and printed copies of all the new clinical resources (e.g. factsheets, discussion tools, client brochures, referrer lists).

*Arm 2. Environmental restructuring* included development of clinical resources and changes to the existing customer management system to assist audiologists with executing the three target behaviours. All of the clinical resources were co-developed with our clinical partner using an iterative process, including pilot testing. Specifically, RJB and three clinical managers from our clinical partner organisation developed the first iteration of the clinical resources (including the client fact sheets, help-seeking lists, brochure on help-seeking pathways, discussion tools, flashcards and the activity scheduling form: see below for details). The first draft was circulated to all members of the research team (including audiology and psychology experts) for iterative revision. The revised versions of the resources were sent to four clinical audiologists from our clinical partner organisation for pilot testing. They were asked to trial using the resources within their consultations, to gain client feedback on the resources, and to report their experiences and recommendations for improving the resources back to the research team. Of note, they were not trained in how to use the resources prior to the piloting but only told the intended purpose of the resources. Feedback from the pilot test was minor and related primarily to language and layout, with one exception. Initially, the research team and clinical management team developed a resource intending to educate GP referral partners. The concept was that each time an audiologist wrote to a GP referral partner, they would include a generic one-page document describing the link between hearing loss and mental wellbeing. Although the research team, clinical managers, and two GPs (colleagues of RJB contacted to review the GP information sheet) all contributed to the development of the GP information sheet and felt that it had merit as a good clinical resource, during the pilot testing, all four clinical audiologists reported that they would not use this resource as they felt it was not appropriate for them to be ‘instructing’ GPs and that sending them may hurt their relationship with GP referrers. The research team reached out to four additional clinical audiologists for feedback on the GP educational resource; all four shared the same concern, stating that they would not use the GP information sheet. Thus, the GP educational resource was removed from the intervention program.

The clinical resources included^1^:

- Client factsheets: describing the social impacts of hearing loss, the emotional impacts of hearing loss, and how hearing loss can impact relationships
- Help-seeking lists: providing lists on where to get help (e.g. lists of local psychologists, socialisation opportunities, mental wellbeing websites)
- A brochure on help-seeking pathways for mental wellbeing
- Discussion tools to promote and normalise discussion about the mental wellbeing impacts of hearing loss and support shared decision making for mental wellbeing help-seeking
- Changes to the customer management system, including
  - Additional questions within the clinical case notes section
  - Modifications to the client rehabilitation goals table
  - Changes to the reports and referrals templates
  - Ability to more easily print out factsheets and client rehabilitation goals
- Flash cards to support self-directed learning about appropriate language when asking about and discussing mental wellbeing
- Activity scheduling form to facilitate encouragement of social re-engagement behaviours
- A client educational video to be routinely sent to clients to help them think about the mental wellbeing impacts of their hearing loss prior to their first appointment at the clinic

*Arm 3. Modelling* was selected to provide an example for staff to aspire to or imitate and to complement the ongoing support elements from arms 1 and 2. Senior and well-respected audiologists from the partner clinic were engaged to record themselves describing which of the new clinical resources were their favourite to use and why, including a demonstration of how they use the tool within their routine workflow. These videos were sent to staff periodically over the 6 months following the roll out of arms 1 and 2.

*The output* of this process was a co-developed, multifaceted behaviour change intervention aiming to improve how audiologists provide mental wellbeing support to adults with hearing loss, the AIMER program, and a context specific implementation strategy.

## Discussion

This paper describes the systematic, structured development of an intervention to improve the frequency of mental wellbeing support provided by audiologists within routine audiological service delivery. The intervention is called AIMER (Ask, Inform, Manage, Encourage and Refer) and is, to our knowledge, the first intervention targeting mental wellbeing support behaviours of audiologists. The AIMER intervention targets three audiologist behaviours: (i) asking clients about their mental wellbeing, (ii) providing general information on the mental wellbeing impacts of hearing loss, and (iii) providing personalised information on managing the mental wellbeing impacts of hearing loss.

Where some interventions target health behaviours at the individual level (e.g. smoking cessation), others target behaviour change in the health practitioner to improve clinical care and, subsequently, client outcomes. Two examples of upskilling allied health professionals in the detection of mental wellbeing needs are the “Post-Stroke Mood Assessment Pathways” intervention, targeting screening for depression and anxiety in the stroke population by social workers [46] and work by Kneebone et al. [47] promoting screening for depression after stroke by occupational therapists within the hospital setting. The target population in these two studies, hospital-based social workers and occupational therapists, was deemed to be the preferred staff member to complete mood screening as their roles within the hospital environment were to improve psychosocial outcomes for in-patients. Thus, barriers to implementing screening protocols were few and mainly related to (i) knowledge regarding validated screening tools, (ii) time, and (iii) environmental issues, including privacy. Consequently, the resultant interventions were able to focus primarily on development of mood assessment pathways and staff training.

In contrast, the target population in the current study, audiologists, was deemed to be the preferred staff member to provide mental wellbeing support to adults with hearing loss by the patient population (adults with hearing loss) [3], yet the audiologists themselves reported mixed feelings about whether they should be the ones to do so [20]. Our preliminary data showed that almost one third of audiologists felt that provision of mental wellbeing support was outside of their scope of practice, two thirds reported feeling under skilled to provide mental wellbeing support, and 10% felt that there was a lack of research evidence indicating that provision of mental wellbeing support was beneficial [20]. As such, development of the AIMER required multiple behaviour change techniques, specifically targeting the capability, opportunity, and motivational factors identified in the earlier stages of this research program. In this way, the AIMER intervention is similar to the CRANIUM intervention (Cardiometabolic Risk Assessment and treatment through a Novel Integration model for Underserved populations with Mental illness) [48], in that it attempts to empower healthcare professionals to take a more active role in the holistic healthcare needs of their clients. Developed using the BCW framework, CRANIUM helps community psychiatrists to expand their focus of practice and take up routine screening for cardiovascular disease, ultimately improving health outcomes for people with mental illness. Utilizing psychiatrists to take on this role represents a significant culture shift and thus required a complex intervention approach to improve communication between mental health and primary care. Similarly, utilising audiologists to identify mental health needs and support help-seeking for the mental wellbeing impacts of hearing loss required a significant culture shift and complex clinician- and clinic-level changes.

The advantages of taking a whole-of-clinic approach to intervention development have been documented by research groups, including in audiology. Ekberg et al. [40] used the BCW to develop an intervention to improve family-centred care in adult audiology services. Involving all clinic staff (audiologists, reception staff, and managers) facilitated development of an organisation-wide shift in culture and behaviours. Similarly, when developing CRANIUM, Mangurian et al. [48] noted the benefits of gaining feedback from multidisciplinary staff during the course of the intervention design to maximise engagement and feasibility of the resultant intervention. We adopted a similar multi-stakeholder approach to Ekberg et al. [40] and Mangurian et al. [48], in that (i) our research team comprised audiology and psychology researchers and clinicians, (ii) the focus of our intervention (target behaviours) were selected and defined by the intended beneficiaries of the intervention (adults with hearing loss) [21], and (iii) during the process of intervention design, we engaged clinical, administration, and managerial staff employed by our partner clinic (implementation site) as well as external referral partners including general practitioners and psychologists.

Bull et al. [49] reported on applying the BCW across multiple teams and multiple sites with a combined intervention. Specifically, clinical teams from paediatrics, midwifery, heart failure, and older adult mental health specialties in four organisations are aiming to change and implement a new model of preventative healthcare. Co-development was reported by stakeholders to be an important perceived mediator of the feasibility of the intervention program and inevitable success of the behaviour change [49], which resonates with the organisational change literature, where there is increasing emphasis on co-development and co-creation of interventions [50]. We, too, followed a participatory approach, seeking stakeholder feedback (involving staff members from our partner clinic) throughout intervention development and implementation planning. This included seeking staff suggestions for intervention components [19, 42], pilot testing of clinical resources prior to implementation, and partnering with key staff members to roll out the intervention components.

Pilot testing was a critical step in developing the AIMER intervention and implementation strategy, helping us to refine elements of the intervention prior to implementation to maximise success within the clinical setting. The benefits of including a small feasibility study in the development process to aid identification of both strengths and weaknesses of the intervention and implementation strategy prior to a larger trial have been described by others. Passey et al. [51] applied the steps of the BCW method to develop an intervention to improve the provision of smoking cessation support in Australian maternity services, the MOHMQuit. They report the inclusion of a pre-testing feasibility study as crucial to intervention success, as pre-testing confirmed that one component of their intervention (targeting midwives) was acceptable and feasible, yet the other component (targeting managers) required bolstering. Specifically, while managers were enthusiastic about the intervention program, they did not engage with many of the roles and activities that were required of them within the intervention roll out. Interviews with managers revealed there were many conflicting priorities and that they were time poor and thus had not engaged with intervention as they would have liked to [51]. Our pilot testing identified potential feasibility issues with the client factsheets developed as part of the intervention, in that audiologists adopted different approaches to printing, storing and providing the factsheets, with many audiologists reporting the effort required to source, print, and store them as being a cause for not using the factsheets at all. Consequently, we engaged IT services to embed an automated printing function within the existing client management software so that audiologists could print out factsheets from a dropdown menu as required.

The BCW has been used to guide development of interventions targeting a wide range of healthcare professionals and their clinical behaviours, and while many use the BCW to guide development of the intervention and implementation strategy simultaneously, others adopt specific implementation frameworks to guide implementation planning in parallel to intervention development. *More Than Meds* is a capacity-building program to enhance pharmacists’ roles in mental health care, developed and implemented through an iterative process drawing on the BCW model, Theoretical Domains Framework and the Consolidated Framework for Implementation Research (CFIR) [52]. Murphy et al. [52] commented on the importance of considering implementation processes during the early stages of intervention development and the value the CFIR to guide implementation planning while simultaneously using the BCW to guide intervention development. At the outset of this project, we opted to adhere to the BCW methodology drawing on the BCW to inform development of both the intervention and the implementation process simultaneously [40, 48, 53], that is, we did not use a specific implementation model or framework to guide implementation planning; instead, we worked with our stakeholders (adults with hearing loss, significant others, clinical audiologists, audiology clinic managers, psychologists, and general practitioners) to design the intervention and mode of delivery concurrently. Although we did not systematically use the RE-AIM framework [54] during implementation planning, we had planned on using the RE-AIM framework to guide our evaluation study and thus were familiar with the RE-AIM components. As a research team, we had discussed making it a specific goal to design the implementation plan so that the intervention components would be embedded within the audiologists’ routine workflow and sustained beyond the life of the research project. Having these principles of the RE-AIM model in our minds while developing the implementation plan likely improved the feasibility of our design. We encourage others to systematically and comprehensively use available implementation models and frameworks to guide implementation planning [55].

### Strengths and challenges of the BCW model

The research team found strengths and challenges in using the BCW for the development of the intervention. The BCW model was selected as it combined 19 health behaviour change models into one comprehensive model for intervention design and implementation planning [32]. A major strength of the BCW model was that it provided a structured step-by-step approach which facilitated a systematic intervention design process, wherein researchers are encouraged to first conduct a deep exploration of the problem behaviour informing selection of simple and specific target behaviours avoiding the attempt to change too many or too complex behaviours at once. The model encourages a high level of involvement with stakeholders across the intervention design process, promoting feasibility of the resultant intervention and implementation approach. Specifically, we began this work with the broad aim of improving mental wellbeing support for adults with hearing loss within the audiological setting. The research team’s presupposition was that the most problematic behaviour preventing adults with hearing loss from receiving mental health support was audiologists not referring their clients to mental health practitioners and general practitioners (family physicians). However, by following the BCW model’s participatory approach, it became apparent that few stakeholders saw referral as an important behaviour [21]. If the systematic process of exploring the problem behaviour from the perspectives of stakeholders had not been executed as a starting point for intervention development within this project, a very different intervention would have evolved that would not have aligned closely with stakeholder needs.

Given the highly systematic and structured approach of the BCW model, the process of intervention development was time-consuming and labour intensive, taking over 12 months to complete all eight steps. While time and resources were accounted for in this instance, the time- and resource-intensive nature of the BCW process may make it unfeasible for certain problems, organisations, or situations. One of the challenges our research team faced was following the comprehensive and lengthy BCW process while trying to cater for the desires of our clinical partners who wanted to implement clinical changes more rapidly. Furthermore, one advantage of the BCW model is that it promotes an in-depth exploration of the barriers and enablers to the target behaviours (guided by the COM-B model), and this process often reveals a long and diverse list of barriers and enablers that need to be addressed. On the other hand, it is often not possible to address all the barriers and potential enablers identified, making it difficult to actualize all the components that can aid in addressing the target behaviours. Additionally, despite the highly comprehensive and systematic approach of the BCW model, there are points in the process where the researcher must make a series of subjective and pragmatic decisions (such as selecting the key barriers and enablers to focus on). This challenge has been noted by other research groups [40, 53], and like them, we used the APEASE criteria to guide decision making. We also ensured that decisions were not made by the research team alone and that our clinical partner representatives were involved in all decision-making processes. As noted by Ekberg et al. [40], we too found that the descriptions of individual behaviour change techniques were sometimes ambiguous and did not always reflect the complexity of what was required to successfully promote behaviour change. It may have been helpful during the design process if the BCW provided more in-depth descriptions of each behaviour change technique and guidance on how they might best be implemented. Also, given that healthcare professional behaviour change lies at the cross-section between behavioural science and implementation science [56], we found that the taxonomy of modes of delivery provided within the BCW was better suited to public health interventions targeting the general public as opposed to healthcare professional behaviour change interventions. Therefore, it might have been more appropriate to draw more on implementation science models to develop our implementation plan such as the Expert Recommendations for Implementing Change ERIC) [57].

Scale-up refers to the replication or expansion of an implementation study to reach more people and/or broaden the effectiveness of an intervention. Given that much of the AIMER intervention was delivered as self-directed learning (through videos), and with only a one-off 3-h in-person online training session, future scale-up of the AIMER program could look to reconfigure all training components to comprise self-directed and or train-the-trainer approaches, much like the recently developed online counselling education program for speech and language pathologists that addresses psychological well-being in those affected by post-stroke aphasia [58].

## Conclusions

Provision of mental wellbeing support in adult audiology services has the potential to improve clinical outcomes and quality of life for adults with hearing loss. This report describes the first study to use the BCW process to develop an intervention targeting mental wellbeing support behaviours in audiologists and confirms the usability and usefulness of the approach in this complex area of clinical care. The AIMER intervention is a three-arm program including (i) a training, education, enablement, and persuasion arm; (ii) an in-house environmental restructuring arm; and (iii) a modelling arm. Pilot testing the intervention allowed for refinement prior to full scale implementation. The systematic, comprehensive, and transparent approach used in the development of the AIMER intervention will facilitate a thorough evaluation of its effectiveness in the next phase of this work, as well as provide an example of the application of the BCW process in this instance, contributing to the knowledgebase for use of the BCW process and the ongoing development and refinement of the model.

Intervention materials can be found at https://osf.io/g2tzu/ or by contacting Dr Bennett (bec.bennett@earscience.org.au).

## Supplementary Information

**Additional file 1.**

**Additional file 2.**

**Additional file 3.**

## Data Availability

Not applicable.

## Abbreviations

AIMER: Ask, Inform, Manage, Encourage, Refer
APEASE: Acceptability, Practicability, Effectiveness, Affordability, Side-effects, and Equity
BCW: Behaviour Change Wheel
CFIR: Consolidated Framework for Implementation Research
COM-B: Capability, Opportunity, Motivation, Behaviour
CRANIUM: Cardiometabolic Risk Assessment and treatment through a Novel Integration model for Underserved populations with Mental illness
ERIC: The Expert Recommendations for Implementing Change
RE-AIM: Reach, Effectiveness, Adoption, Implementation, and Maintenance
TIDieR: The Template for Intervention Description and Replication

## Footnotes

1 Copies of the clinical resources can be found at https://osf.io/g2tzu/
