# Supplementary material for: Use of the Behaviour Change Wheel to design an intervention to improve the provision of mental wellbeing support within the audiology setting
Source: Implement Sci Commun. 2023 May 2;4:46. doi: 10.1186/s43058-023-00427-1 (PMC10153035; doi:10.1186/s43058-023-00427-1)
Supplement: Supplementary file 1 — Additional file 1. [file 43058_2023_427_MOESM1_ESM.docx]

***Additional File One.*** All 93 factors (barriers and facilitators) identified across all three target behaviours in the COM-B analysis (Step 4; Adapted from Nickbakht et al, 2022 and Bennett et al, 2022), whether they selected to form the basis of the final intervention (n=19) and reasons for non-selection.

| **COM-B domain** | **Factors identified through the COM-B analysis** | **Barrier/facilitator/mixed (number of statements describing this phenomenon)** | **Target Behaviour*** | **Wording of target barrier/facilitator if selected (as some combined)** | **Reason for non-selection** |
| --- | --- | --- | --- | --- | --- |
| Physical Capability (Physical skill, strength or stamina) | | |  |  |  |
|  | N/A | N/A | Ask, Inform, Manage | N/A | N/A |
| Psychological Capability (Knowledge or psychological skills, strength or stamina to engage in the necessary mental processes) | | | |  |  |
|  | No knowledge of how to detect the signs and symptoms for emotional distress | Barrier (3) | Ask | Audiologists require knowledge of how to detect signs and symptoms for emotional and psychological distress |  |
|  | Knowledge of mental health signs and symptoms | Facilitator (2) | Manage |  |  |
|  | (No) Knowledge of how to approach/ask about emotional well-being | Mixed (8) | Ask | Audiologists require knowledge of how to ask about emotional well-being |  |
|  | Not knowing the triggers to ask | Barrier (1) | Ask |  |  |
|  | Not having the language to ask about emotional well-being | Barrier (2) | Ask |  |  |
|  | (No) counselling skills | Mixed (5) | Ask |  |  |
|  | Knowing about resources/where to find resource | Facilitator (2) | Ask |  | Clinicians demonstrated a good knowledge base |
|  | Not knowing how to measure psychosocial wellbeing | Barrier (1) | Ask |  | Not currently part of clinical processes; not vital |
|  | Building rapport/trust with clients | Facilitator (2) | Ask |  | Clinicians demonstrated a good knowledge base |
|  | (Lack of) skill of getting deep about emotional well-being | Mixed (2) | Ask |  | Advanced skill. We decided to work on the basic skills first and will look into advanced training for clinicians as a secondary study |
|  | Asking about emotional well-being is difficult | Mixed (2) | Ask |  | * Linked to **Psychological Capability**. Audiologists require knowledge of how to ask about emotional well-being and **Psychological Capability**. Audiologists require language skills for discussing mental health-related topics |
|  | Forgetting to ask | Barrier (2) | Ask |  | * Linked to **Automatic Motivation**. Audiologists need reminders/prompts to help them remember to ask clients about mental wellbeing |
|  | Forgetting to provide information on the emotional impacts of hearing loss during client interactions | Barrier (1) | Inform |  |  |
|  | Audiologists’ awareness of emotional impacts of hearing loss | Facilitator (32) | Ask |  | Clinicians demonstrated a good knowledge base |
|  | (No) knowledge of where to access additional information and resources | Mixed (5) | Inform |  | This was generally not a barrier for clinicians |
|  | Lack of language skills for describing and discussing the social and emotional impacts of hearing loss | Barrier (3) | Inform | Audiologists require language skills for discussing mental health-related topics |  |
|  | Skills for discussing mental health-related topics | Facilitator (4) | Manage |  |  |
|  | (No) knowledge of mental wellbeing management options | Mixed (19) | Manage | Audiologists require knowledge of treatment/management options for emotional and psychological distress, and how to provide reliable information regarding funding and access for psychological services |  |
|  | No knowledge of professional services / funding / access | Barrier (15) | Manage |  |  |
|  | (Lack of) language skills to discuss or describe mental health support options | Mixed (10) | Manage |  |  |
|  | No knowledge of mental health self-help strategies | Barrier (2) | Manage |  |  |
|  | Knowledge of mental health programmes / techniques delivered by psychologists | Facilitator (8) | Manage |  |  |
|  | Knowledge of how to find information as required | Facilitator (2) | Manage |  |  |
|  | No knowledge of Guidelines | Barrier (7) | Manage |  | No guidelines currently available for audiologists |
|  | No knowledge of resources / tools to aid discussion of options | Barrier (5) | Manage |  | No shared-decision making resources currently available that consider mental wellbeing needs |
|  | No knowledge of which approaches are within/outside of the HHCs' scope of practice | Barrier (2) | Manage |  | * Linked to **Reflective Motivation**. Audiologists need reassurance that asking about and providing mental wellbeing support is within their scope of practice |
|  | (No) knowledge of referral processes, who to refer to or how to refer | Mixed (23) | Manage | Audiologists require knowledge of who to refer to and how to refer for mental wellbeing support |  |
|  | Lack of knowledge regarding which clients are likely to benefit from mental health support, and by how much | Barrier (1) | Manage |  | A complex phenomenon and difficult to teach within the time constraints of this project |
|  | Inability to execute clinical procedures due to a lapse in skills | Barrier (5) | Manage |  | The need for ongoing training and support was not incorporated into the intervention per say, but into the implementation strategy |
|  | Inability to apply person-centred care | Barrier (1) | Manage |  | This was generally not a barrier for clinicians |
| Physical Opportunity (Opportunity afforded by the environment involving time, resources, locations, cues, physical ‘affordability’) | | | |  |  |
|  | Time for asking about emotional well-being | Mixed (7) | Ask |  | Time was generally not a barrier for clinicians |
|  | (Lack of) time | Mixed (5) | Inform |  |  |
|  | (Lack of) time | Mixed (6) | Manage |  |  |
|  | Lack of Tools (e.g., clinical resources) | Barrier (2) | Ask | Audiologists require clinical resources to assist with (i) asking about wellbeing, (ii) providing information on the wellbeing impacts of hearing loss, and (iii) providing information on wellbeing treatment/management strategies |  |
|  | Lack of internal resources | Barrier (2) | Inform |  |  |
|  | Availability of resources from external organisations | Facilitator (2) | Inform |  |  |
|  | (Lack of) clinical resources aiding provision of mental health support | Mixed (18) | Manage |  |  |
|  | Financial barriers | Barrier (7) | Manage |  | Time was generally not a barrier for clinicians |
|  | Lack of mental health services appropriate for people with hearing loss | Barrier (6) | Manage |  | Although this barrier is likely to have an impact on the target behaviours, it was deemed to be too difficult to address within the project timeframe |
| Social Opportunity (Opportunity afforded by interpersonal influences, social cues and cultural norms that influence the way we think about things) | | | |  |  |
|  | (Lack of) clients’ openness to questions about emotional well-being | Mixed (26) | Ask | Audiologists require reassurance that clients will be receptive to them asking about and providing mental wellbeing |  |
|  | Clients are (not) open to receiving information on the emotional impacts of hearing loss | Mixed (13) | Inform |  | * Linked to **Reflective Motivation**. Audiologists require reassurance that clients are open to receiving information on mental wellbeing treatment/management options during audiological appointments |
|  | Clients are not open to discussing mental health support options | Barrier (11) | Manage |  |  |
|  | Clients create opportunity and are open to discussing mental health support | Facilitator (9) | Manage |  |  |
|  | If clients bring it up, audiologist asks about emotional impacts of hearing loss | Facilitator (4) | Ask |  | This was generally not a barrier for clinicians |
|  | Cochlear implant clients more willing to express feelings than hearing aid clients | Facilitator (3) | Ask |  | This was generally not a barrier for clinicians |
|  | Male clients do not feel comfortable talking about emotions | Barrier (2) | Ask |  | This was generally not a barrier for clinicians |
|  | Lack of client awareness about audiologists’ role in supporting emotional wellbeing | Barrier (3) | Ask |  | *Linked to **Social Opportunity**. Audiologists require reassurance that clients will be receptive to them asking about mental wellbeing |
|  | Supportive Peers | Facilitator (5) | Ask | Audiologists need to feel supported by peers in their workplace |  |
|  | Working in an environment with supportive colleagues who encourage the provision of information relating to the emotional impacts of hearing loss | Facilitator (4) | Inform |  |  |
|  | Normalisation of discussions relating to emotional well-being (reduced stigma) | Facilitator (3) | Ask | Audiologists need to see their managers and senior staff role modelling provision of mental wellbeing support |  |
|  | Working in an environment with supportive managers who encourage the provision of information relating to the emotional impacts of hearing loss | Facilitator (4) | Inform | Audiologists require reassurance from their managers that provision of mental wellbeing support is a vital part of their service provision despite it not being a claimable service. |  |
|  | Experienced and supportive managers and colleagues who role model best-practice | Mixed (11) | Manage |  |  |
|  | Presences of significant others | Facilitator (5) | Ask |  | This was generally not a barrier for clinicians |
|  | Clients provide opportunities to discuss the emotional impacts of hearing loss, and even raise the topic | Facilitator (1) | Inform |  | This was generally not a barrier for clinicians |
| Automatic Motivation (Automatic processes involving emotional reactions, desires, impulses, inhibitions, drive states and reflex responses) | | | | | |
|  | Feeling comfortable (or not) with asking clients about wellbeing | Mixed (10) | Ask |  | The need for audiologists to overcome their negative emotions preventing them from providing mental wellbeing support were initially deemed an important component of the intervention; however, during the process of intervention development we had to eliminate some elements to manage time constraints and this factor was dropped. |
|  | Anticipated regret about asking clients about wellbeing | Barrier (1) | Ask |  |  |
|  | Feeling anxious about asking clients about wellbeing | Barrier (1) | Ask |  |  |
|  | Feeling helpless due to not knowing how to fully support clients | Barrier (3) | Manage |  |  |
|  | Feeling uncomfortable when discussing mental health management options with clients | Barrier (7) | Manage |  |  |
|  | Being in the habit of asking clients about wellbeing | Mixed (6) | Ask | Audiologists need reminders/prompts to help them remember to ask clients about mental wellbeing |  |
|  | Use of reminders/prompts to promote asking clients about wellbeing | Facilitator (4) | Ask |  |  |
|  | Reinforcement through clinic policy | Facilitator (1) | Inform |  | Although this was not included as part of the intervention per say, this factor was considered when developing the implementation strategy |
|  | Desire to develop and provide information via written resources | Facilitator (1) | Inform |  | Clinicians' motivations toward providing mental wellbeing support were generally not a barrier |
|  | Happy to provide information | Facilitator (3) | Inform |  |  |
|  | Feeling good when able to help clients | Facilitator (4) | Manage |  |  |
|  | Motivated to help clients to avoid future negative feelings | Facilitator (4) | Manage |  |  |
|  | Fears clients' potential negative reactions to mental health related discussions | Barrier (12) | Manage |  | * Linked to **Reflective Motivation**. Audiologists require reassurance that clients are open to receiving information on mental wellbeing treatment/management options during audiological appointments |
|  | Lacks confidence in ability to refer to appropriate mental health specialists | Barrier (3) | Manage |  | *Linked to **Psychological Capability**. Audiologists require knowledge of who to refer to and how to refer for mental wellbeing support |
|  | Confidence in own ability to provide mental health support | Facilitator (2) | Manage |  | *Linked to **Psychological Capability**. Audiologists require knowledge of treatment/management options for emotional and psychological distress, and how to provide reliable information regarding funding and access for psychological services |
|  | Emotionally comfortable with providing mental health support | Facilitator (2) | Manage |  |  |
| Reflective Motivation (Reflective processes involving planning and evaluation) | | | |  |  |
|  | Beliefs about consequences from asking clients about wellbeing | Mixed (15) | Ask | Audiologists need to feel responsible for (i) asking about mental wellbeing, (ii) providing information on the mental wellbeing impacts of hearing loss, and (iii) providing information on mental wellbeing treatment/management strategies |  |
|  | Intention to prioritise and share information | Facilitator (4) | Inform |  |  |
|  | Beliefs about positive impacts/benefits | Facilitator (7) | Inform |  |  |
|  | Doubts about whether mental health services are truly beneficial for clients with hearing loss | Barrier (8) | Manage |  |  |
|  | Beliefs that addressing mental health results in improved client outcomes | Facilitator (22) | Manage |  |  |
|  | Belief that providing mental health support enhances audiological practice | Facilitator (11) | Manage |  |  |
|  | A desire to provide mental health support | Facilitator (12) | Manage |  |  |
|  | A desire to learn how to better provide mental health support | Facilitator (5) | Manage |  |  |
|  | Optimistic about the benefits of providing mental health support | Facilitator (2) | Manage |  |  |
|  | Beliefs about confidence/capabilities for asking clients about wellbeing | Mixed (11) | Ask | Audiologists need to develop confidence in their ability to ask about mental wellbeing and respond with empathy when clients describe their challenges |  |
|  | (Lack of) Confidence | Mixed (7) | Inform |  |  |
|  | Personal interest in the client as an individual | Facilitator (11) | Ask |  |  |
|  | Beliefs about whether asking clients about wellbeing is with in the scope of audiology practice | Mixed (5) | Ask | Audiologists need reassurance that asking about and providing mental wellbeing support is within their scope of practice |  |
|  | Beliefs about clinical boundaries with respect to the HHCs scope of practice | Mixed (20) | Manage |  |  |
|  | Limiting beliefs concerning the role and responsibility of the HHC | Barrier (6) | Manage |  |  |
|  | Belief that clients would react unfavourably if provided information on mental wellbeing | Barrier (4) | Inform | Audiologists require reassurance that clients are open to receiving information on mental wellbeing treatment/management options during audiological appointments |  |
|  | Belief that clients would react unfavourably if the audiologist provided advice on managing mental wellbeing | Barrier (4) | Manage | Audiologists require reassurance that clients are open to receiving information on mental wellbeing treatment/management options during |  |
|  | Belief that clients are open to discussing mental health management options | Facilitator (5) | Manage |  |  |
|  | Beliefs about GPs perceptions regarding the role of the HHCs in providing mental health support | Mixed (6) | Manage | Audiologists need reassurance that GPs would react positively to receiving a referral from an audiologist regarding concerns for a client’s mental wellbeing |  |
|  | Beliefs about psychologists' ability to address the needs of adults with hearing loss seeking mental health support | Barrier (8) | Manage | Audiologists need reassurance that psychologists have the skills required to address the psychological needs of adults with hearing loss seeking psychological support |  |
|  | Beliefs about psychologists openness to receiving referrals from HHCs | Facilitator (3) | Manage | Audiologists need reassurance that psychologists are open to receiving referrals from them |  |
|  | Beliefs about the need to find the right mental health practitioner to suit the client’s needs | Facilitator (4) | Manage |  | Clinicians' motivations towards working more collaboratively with mental health practitioners was generally not a barrier |
|  | Desire to work more collaboratively with mental health services | Facilitator (7) | Manage |  |  |

*Three target behaviours (i) Ask: asking clients about their mental wellbeing; (ii) Inform: providing general information on the mental wellbeing impacts of hearing loss; and (iii) Manage: providing personalised information on managing the mental wellbeing impacts of hearing loss.
