# Supplementary material for: Use of the Behaviour Change Wheel to design an intervention to improve the provision of mental wellbeing support within the audiology setting
Source: Implement Sci Commun. 2023 May 2;4:46. doi: 10.1186/s43058-023-00427-1 (PMC10153035; doi:10.1186/s43058-023-00427-1)
Supplement: Supplementary file 3 — Additional file 3. [file 43058_2023_427_MOESM3_ESM.docx]

***Additional File Three.*** Identification of intervention functions (Step 5)

| **COM-B domain** | **Barriers/facilitators selected** | **Target behaviour*** | **Possible intervention functions**  (selected intervention functions bolded) |
| --- | --- | --- | --- |
| **Physical Capability** (Physical skill, strength or stamina) | | | |
|  | N/A |  |  |
| **Psychological Capability** (Knowledge or psychological skills, strength or stamina to engage in the necessary mental processes) | | | |
|  | **Psychological Capability.** Audiologists require knowledge of how to detect signs and symptoms for emotional and psychological distress | Ask | **Training** |
|  | **Psychological Capability.** Audiologists require knowledge of how to ask about emotional well-being | Ask | **Training** |
|  | **Psychological Capability.** Audiologists require language skills for discussing mental health-related topics | Inform & Manage | **Training** |
|  | **Psychological Capability.** Audiologists require knowledge of treatment/management options for emotional and psychological distress, and how to provide reliable information regarding funding and access for psychological services | Manage | **Training** |
|  | **Psychological Capability.** Audiologists require knowledge of who to refer to and how to refer for mental wellbeing support | Manage | **Education**  **Environmental restructuring**  **Training** |
| **Physical Opportunity** (Opportunity afforded by the environment involving time, resources, locations, cues, physical ‘affordability’) | | | |
|  | **Physical Opportunity.** Audiologists require clinical resources to assist with (i) asking about wellbeing, (ii) providing information on the wellbeing impacts of hearing loss, and (iii) providing information on wellbeing treatment/management strategies | Ask, Inform & Manage | Training  Restriction  **Environmental restructuring**  Enablement |
| **Social Opportunity** (Opportunity afforded by interpersonal influences, social cues and cultural norms that influence the way we think about things) | | | |
|  | **Social Opportunity.** Audiologists require reassurance that clients will be receptive to them asking about mental wellbeing | Ask | Education  **Environmental restructuring**  Modelling Enablement |
|  | **Social Opportunity.** Audiologists require reassurance from their managers that provision of mental wellbeing support is a vital part of their service provision despite it not being a claimable service. | Ask, Inform & Manage | Restriction  **Environmental restructuring**  Modelling Enablement |
|  | **Social Opportunity.** Audiologists need to feel supported by peers in their workplace | Ask, Inform & Manage | Restriction **Environmental restructuring** Modelling  **Enablement** |
|  | **Social Opportunity.** Audiologists need to see their managers and senior staff role modelling provision of mental wellbeing support | Ask, Inform & Manage | Restriction Environmental restructuring **Modelling**  Enablement |
| **Automatic Motivation** (Automatic processes involving emotional reactions, desires, impulses, inhibitions, drive states and reflex responses) | | | |
|  | **Automatic Motivation.** Audiologists need reminders/prompts to help them remember to ask clients about mental wellbeing | Ask | **Training** Incentivisation  Coercion  **Environmental restructuring** |

| **Reflective Motivation** (Reflective processes involving planning and evaluation) | |  |  |
| --- | --- | --- | --- |
|  | **Reflective Motivation.** Audiologists need to develop confidence in their ability to ask about mental wellbeing and respond with empathy when clients describe their challenges | Ask & Inform | **Training**   Modelling Enablement |
|  | **Reflective Motivation.** Audiologists need reassurance that clients are open to receiving information on the mental wellbeing impacts of hearing loss | Inform | **Education & Persuasion** |
|  | **Reflective Motivation.** Audiologists need reassurance that asking about and providing mental wellbeing support is within their scope of practice | Ask & Manage | **Education**  Persuasion Modelling |
|  | **Reflective Motivation.** Audiologists need to feel responsible for (i) asking about mental wellbeing, (ii) providing information on the mental wellbeing impacts of hearing loss, and (iii) providing information on mental wellbeing treatment/management strategies | Ask, Inform & Manage | Education  **Persuasion**  Modelling |
|  | **Reflective Motivation.** Audiologists require reassurance that clients are open to receiving information on mental wellbeing treatment/management options during audiological appointments | Inform & Manage | Education  **Persuasion**  Modelling |
|  | **Reflective Motivation.** Audiologists need reassurance that GPs would react positively to receiving a referral from an audiologist regarding concerns for a client’s mental wellbeing | Manage | Education  **Persuasion**  Modelling |
|  | **Reflective Motivation.** Audiologists need reassurance that psychologists have the skills required to address the psychological needs of adults with hearing loss seeking psychological support | Manage | Education  **Persuasion**  Modelling |
|  | **Reflective Motivation.** Audiologists need reassurance that psychologists are open to receiving referrals from them | Manage | Education  **Persuasion**  Modelling |
